# Phytochemical Composition, Biological Activities, and Mechanisms of Antibacterial Action of Selected Cameroonian Medicinal Plants

**DOI:** 10.7759/cureus.86251

**Published:** 2025-06-17

**Authors:** Germaine Takongmo Matsuete, Benjamin Tangue Talom, Jean-De-Dieu Tamokou

**Affiliations:** 1 Department of Biochemistry, University of Dschang, Dschang, CMR; 2 Department of Biomedical Sciences, University of Ngaoundere, Ngaoundere, CMR

**Keywords:** antibacterial, antioxidant, dehydrogenase activity, medicinal plants, membrane permeability, phytochemistry, synergy

## Abstract

Background

Medicinal plants have always played a very important role in the control and prevention of diseases, especially those caused by antibiotic-resistant bacteria. This study aimed to evaluate the antibacterial activity, antioxidant potential, synergistic effect, phytochemical content, and mechanisms of antibacterial action of the selected Cameroonian medicinal plants.

Methodology

The extracts were prepared by maceration in ethanol. The antibacterial activities of the extracts alone and the combinations of extracts with Augmentin were determined by the microdilution method in a liquid medium. The mode of action of plant extracts was studied by targeting bacterial ATPases/H^+^ proton pump function, outer membrane permeability, dehydrogenase activity, and leakage of nucleic acids. The antioxidant activities and phytochemical analysis were evaluated using colorimetric methods.

Results

All the crude extracts tested showed different degrees of antibacterial activities (minimum inhibitory concentration (MIC) = 64-2048 µg/ml) on Gram-positive and negative bacteria, which can be linked to their total phenolic, flavonoid, sterol, triterpene, and tannin contents. *Kotschya strigosa* leaf extract was the most active tested sample and exerted, with Augmentin, additive, synergistic, and indifferent effects on bacterial species. *K. strigosa* leaf extract induced changes in the permeability of bacterial membranes, leakage of the nucleic acids, and the inhibition of the bacterial ATPases/H^+^ proton pump. *K. strigosa* and *Kalanchoe pinnata* extracts displayed potent antioxidant (EC_50_ = 2.20-2.24 μg/ml; ferric-reducing antioxidant power = 142.58-142.67 μg/ml) activity.

Conclusions

The results of this study demonstrated the antibacterial and antioxidant activities of the tested plants and confirmed their traditional use in the treatment of infectious diseases caused by the tested bacteria.

## Introduction

Globally, acute respiratory infections remain a leading cause of morbidity, hospitalization, and mortality in people of all ages [1]. Data from a systematic review show that in 2016, approximately 336 million episodes in people of all ages resulted in more than 65 million hospitalizations, with an overall mortality of 2.3 million deaths. Some of these diseases are called chronic, while others are acute, such as pneumonia [2], which causes more than 17 million deaths per year worldwide, more than half of which occur on the African continent alone [3]. Respiratory diseases, with a rate of 14%, rank second among the pathologies responsible for more deaths in Cameroon [2]. These respiratory infections are often caused by bacteria, so the most striking example to illustrate some infections is* Klebsiella pneumoniae*, first described by Carl Friedlander in 1882 as a bacterium isolated from the lungs of patients who died of pneumonia [4]. Current treatment of *K. pneumoniae* infections is limited due to antibiotic tolerance and multi-resistance of strains due to the acquisition of genetic characteristics, namely the production of broad-spectrum β-lactamase enzymes, leading to failure of antimicrobial treatment, recognized as a global crisis in modern medicine [5]; these infections also have a high cost, both for the families of the sick and for society in general, in terms of temporary disability, school absenteeism, and loss of workdays [2]. The damage caused by these conditions continues to increase with pollution and the resistance of infectious agents to generic drugs. This is why the World Health Organization (WHO) has classified *K. pneumoniae* as one of the priority and critical pathogens that require urgent research and development of new and effective antibiotics [6]. Plant sources used since ancient times to fight microbial infections appear as an interesting alternative for the discovery of new antibacterial substances against multidrug-resistant bacteria [7]. Higher plants produce hundreds of thousands of diverse chemical compounds with different biological activities and important ecological roles. In many cases, these substances or compounds serve as a chemical defense mechanism against insects, herbivores, and microorganisms. In vitro and epidemiological studies suggest that the consumption of foods rich in phenolic compounds could significantly reduce the risk of certain health problems due to their antioxidant and antibacterial properties [8]. Hence, this study aimed to evaluate the antibacterial activity, the synergistic effect, the phytochemical content, and the mode of action of the selected Cameroonian medicinal plants.

## Materials and methods

Plant collection and extract preparation

The leaves/fruits of eight plant species were harvested in September 2022 in the cities of Ebolowa and Dschang (Table 1). These plants were chosen on the basis of their traditional use in the treatment of bacterial infections. The plants were identified and authenticated at the Cameroon National Herbarium, Cameroon, where the voucher specimens were kept under the reference numbers (Table 1). The leaves/fruits of each plant species were dried separately at room temperature (20-25°C) and protected from light until a constant weight was obtained, then ground into powder in a mixer (Table 1). The powder obtained after grinding was macerated in ethanol for 48 hours with occasional shaking to maximize the yield. After 48 hours, the mixture was filtered using Whatman No. 1 paper, and the filtrate obtained was evaporated using a rotary evaporator at 78°C. The extract obtained was collected in a dry and sterile bottle and then introduced into an oven at 40°C until the solvent had completely evaporated. The extraction yield for each plant species was calculated relative to the mass of the dry plant powder.

**Table 1:** Scientific name, plant parts used, and voucher specimen number of the studied medicinal plants HNC: Camerouon National Herbarium; SRF/Cam: *Section de réserve forestière du Cameroun *(Cameroon Forest Reserve Section)

Scientific name (family)	Part used	Voucher specimen number
*Kotschya strigosa* (Benth) (Mimosoideae)	Leaves	22849/SRF/Cam
*Eucalyptus globulus *(Myrtaceae)	Leaves	1868/SRF/Cam
*Kalanchoe pinnata* (Crassulaceae)	Leaves	35196/HNC
*Piper umbellatum* (Piperaceae)	Leaves	44113/HNC
*Eupatorium cannabinum* (Asteraceae)	Leaves	8629/HNC
*Annona muricata* (Annonaceae)	Leaves	32879/HNC
*Sapindus saponaria* (Sapindaceae)	Fruits	15735/HNC
*Abelmoschus esculentus* (Malvaceae)	Leaves	8537/SRF/Cam

Chemical analysis of extracts

Qualitative Phytochemical Analysis of Extracts

The main classes of compounds (alkaloids, flavonoids, anthraquinones, tannins, saponins, anthocyanins, triterpenes, phenols, polyphenols, and sterols) were detected in the extracts as previously described [9].

Quantitative Phytochemical Analysis of Extracts

The total phenol content was determined as previously described [10]. A calibration curve was plotted using gallic acid (gallic acid concentration ranged from 0.015 mg/mL to 2 mg/mL). The results were expressed as milligrams equivalent of gallic acid per gram of extract. The total flavonoid content of the extracts was determined using the aluminum chloride colorimetric method [11]. The absorbance was measured by a spectrophotometer at 415 nm. The total flavonoid content was calculated using the quercetin calibration curve (quercetin concentration ranged from 0.015 mg/mL to 2 mg/mL), and the results were expressed as milligrams equivalent of quercetin per gram of extract. The total tannin content was determined by the Folin-Ciocalteu method [12]. The absorbance was measured by a spectrophotometer at 700 nm. A calibration curve was plotted using different concentrations (100, 200, 300, 400, 500 µg/mL) of tannic acid. The results were expressed as milligrams equivalent of tannic acid per gram of extract.

Antibacterial assays

Microorganisms

The bacteria used in this work consisted of clinical isolates and reference strains from the American Type Culture Collection (ATCC) of *K. pneumoniae*, *Pseudomonas aeruginosa,* and *Streptococcus pneumoniae*. These bacterial species were preserved in the Laboratory of Microbiology and Antimicrobial Substances in a mixture of glycerol and Mueller-Hinton broth (MHB) (1:1) at -4°C, and activation was done with the streak technique on agar medium prior to any antibacterial test.

Preparation of Bacterial Inocula

The inocula of bacteria was prepared from overnight cultures [13]. The desired bacterial concentration for the test was 10^6^ CFU/mL.

Determination of Minimum Inhibitory Concentrations (MICs) and Minimum Bactericidal Concentrations (MBCs)

The inhibitory potential of bacterial growth of plant extracts was determined by the microdilution method as described [13]. Sample solutions were prepared in 10% aqueous dimethyl sulfoxide (DMSO, Fisher Chemicals, Strasbourg, France) at a concentration of 4096 µg/mL and serially diluted in MHB. Amoxicillin (Sigma Aldrich, Sternheim, Germany) and Augmentin (amoxicillin/clavulanic acid (8/1), Sigma Aldrich, Sternheim, Germany) were used as reference antibiotics. Bacterial growth was monitored colorimetrically using iodonitrotetrazolium chloride (INT). The MIC was the lowest concentration of plant extracts that prevented the change of color. The MBCs were obtained by subculture on Mueller-Hinton agar (MHA) medium of 10 µL of the contents of the wells that did not show visible growth to the naked eye. The lowest concentrations that induced an absence of colonies or the appearance of a colony only on the subculture dishes were considered MBC. Three repetitions were carried out per test solution and per concentration. A plant extract with an MBC/MIC ratio less than or equal to 4 (≤4) was described as bactericidal, while an extract with an MBC/MIC ratio strictly greater than 4 (˃4) was considered bacteriostatic [13].

Evaluation of the Antibacterial Activity of the Associations Between the Extracts and Antibiotic

The checkerboard microdilution method was used to evaluate the association between Augmentin and the most active extract [14]. The fractional inhibitory concentration (FIC) index for combinations of two antibacterial agents was calculated according to the following formula: \[\text{FIC Index} = \text{FIC}_A + \text{FIC}_B\] where \[ \text{FIC}_A = \frac{\text{MIC of Augmentin in combination with extract}}{\text{MIC of Augmentin alone}} \] and \[ \text{FIC}_B = \frac{\text{MIC of extract in combination with Augmentin}}{\text{MIC of extract alone}} \] The FIC indices were interpreted as follows: ≤ 0.5 corresponds to synergy; 0.5 < FIC ≤ 1 corresponds to addition; > 1 < FIC ≤ 2 corresponds to indifference and FIC > 2.0 corresponds to antagonism. All the experiments were performed in triplicate.

Antibacterial Mechanism Studies

The inhibition of the bacterial ATPases/H+ proton pump, outer membrane permeability, dehydrogenase activity, and leakage of nucleic acid assays were used to determine the mode of antibacterial action.

Permeability Assay of the Outer Membrane to Erythromycin

The potentiating effect on the membrane permeability to erythromycin was carried out as described earlier [15]. Bacterial growth was measured in a microplate reader (FLUOstar Omega microplate reader, BMG LABTECH GmbH, Germany) at 450 nm. The test was carried out in triplicate.

Determination of Bacterial Growth Kinetics

To assess the effect of crude extracts on bacterial growth kinetics, the optical densities (ODs) were measured following the protocol previously described [7]. These inocula were treated with the extract at concentrations of ½ MIC, 1xMIC, and 2xMIC and incubated under shaking at a speed of 130 rpm using a magnetic stirrer to allow good dispersion. Vials containing Augmentin served as positive control and those containing MHB + *K. pneumoniae* suspension served as negative control. After several incubation times corresponding to t = 0 minutes, 30 minutes, 1 hour, 2 hours, 4 hours, 6 hours, 8 hours, 10 hours, 12 hours, 14 hours, 16 hours, and 18 hours, 200 μL of each solution were introduced into the wells of flat-bottomed microplates and the ODs were read at 600 nm. Each test was repeated three times.

*Determination of the Enzymatic Activity of Respiratory Chain Dehydrogenases in *K. pneumoniae

The activity of dehydrogenases was evaluated using the colorimetric method [15]. Under physiological conditions, colorless INT is reduced by the bacterial respiratory chain dehydrogenase to a dark-red water-insoluble iodonitrotetrazolium formazan (INF); thus, the dehydrogenase activity can be determined by the change in the spectrophotometric value of INF. Augmentin was used as a positive control. A negative control containing *K. pneumoniae* cultures was boiled for 20 minutes to completely inactivate the enzymes, while unboiled cultures whose enzymes maintained their native activity were considered as the control (+). The culture was centrifuged to collect the bacteria and 250 μL of acetone-ethanol mixture 1:1 (v/v) was used to distill the INF twice. The resulting supernatant was finally collected and the dehydrogenase activity was then calculated based on the maximum spectrophotometric absorbance of INF at 490 nm on a microplate reader.

H+/ATPase Proton Pump Assay

The effects of the ethanolic extract on H+/ATPase proton pumps were carried out by controlling the acidification of the bacterial growth medium using the protocol previously described [15].

Loss of Absorbing Material at 260 nm

Nucleic acid leakage was used as an indicator of major membrane damage and monitored in this study according to the early described method [15]. The assay was performed in triplicate.

Antioxidant assays

Diphenyl-1-Picrylhydrazyl (DPPH) Free Radical Scavenging Assay

The DPPH test of the samples was evaluated as described earlier [13]. The ODs were read at 517 nm using a spectrophotometer (FLUOstar Omega Microplate Reader). The EC_50_ (μg/mL) represents the amount of the sample that can trap 50% of DPPH. All the experiments were done in independent replicates. Vitamin C (L-ascorbic acid) was used as a positive control.

Ferric-Reducing Antioxidant Power (FRAP) Assay

The reducing power of the samples was determined according to the described protocol [16]. Vitamin C was used as a positive control. The antioxidant power of the sample was calculated from the calibration curve of the FeSO_4_ solution (the number of moles of the FeSO_4_ solution ranging from 156.25 µmol to 10000 µmol) and expressed in micromole equivalent FeSO_4_ per gram of sample.

Statistical analysis

The data were expressed as mean ± standard deviation (SD). Statistical analysis was performed using one-way analysis of variance (ANOVA) with post hoc Waller-Duncan’s multiple range tests with IBM SPSS Statistics for Windows, Version 23 (Released 2015; IBM Corp., Armonk, New York, United States). A p-value less than 0.05 was considered significant.

## Results

Chemical analysis of the plant extracts

Flavonoids, phenols, sterols, triterpenes, and tannins were detected in all the tested extracts (Table 2). However, anthocyanins were detected only in the extract of *Kalanchoe pinnata* (leaves); saponins were found in all the tested plants; alkaloids were present only in the extracts of *K. pinnata* (leaves), *Piper umbellatum*, and* Eucalyptus globulus* (leaves); and anthraquinones were not detected in the extracts of *Eupatorium cannabinum* (leaves), *Sapindus saponaria*, and *Abelmoschus esculentus* (leaves). The phenolic, flavonoid, and tannin contents varied depending on the plant species and the part used (Table 3). Hence, the extract of *E. globulus* leaf extract had the highest content of phenolic compounds, whereas *A. esculentus* leaf extract had the lowest value of phenolic compounds. *K. pinnata* and *E. globulus *(leaves) extracts presented the highest amounts of total flavonoid and tannin, respectively.

**Table 2:** Main groups of compounds present in the studied plant extracts -: absence; +: presence

Main groups of compounds	Kotschya strigosa	*Kalanchoe pinnata* (leaves)	Piper umbellatum	*Eupatorium cannabinum *(leaves)	Sapindus saponaria	*Eucalyptus globulus *(leaves)	*Annona muricata* (leaves)	*Abelmoschus esculentus* (leaves)
Alkaloids	-	+	+	-	-	+	-	-
Phenols	+	+	+	+	+	+	+	+
Flavonoids	+	+	+	+	+	+	+	+
Sterols	+	+	+	+	+	+	+	+
Triterpenes	+	+	+	+	+	+	+	+
Tannins	+	+	+	+	+	+	+	+
Saponins	+	+	+	+	+	+	+	+
Anthocyanins	-	+	-	-	-	-	-	-
Anthraquinones	+	+	+	-	-	+	+	-

**Table 3:** Total phenol, flavonoid, and tannin contents of tested plant extracts Each value represents the mean ± standard deviation of three repetitions; in the same column, the values ​​assigned different letters (a-g) are significantly different at the 5% probability level. TPT: total phenol content; TFT: total flavonoid content; TTT: total tannin content; EAG: gallic acid standard; EQ: quercetin standard; EAT: tannic acid standard

Samples	TPT (mg EAG/g of extract)	TFT (mg EQ/g of extract)	TTT (mg EAT/g of extract)
Kotschya strigosa	47.36 ± 0.12^c^	10.08 ± 0.18^a, b^	3.08 ± 0.66^a^
Kalanchoe pinnata	40.36 ± 0.26^a, b^	11.70 ± 0.67^b^	3.18 ± 0.86^a^
Piper umbellatum	108.6 ± 0.06^e^	8.92 ± 0.52^a^	17.60 ± 0.5^d^
*Eupatorium cannabinum* (leaves)	43.93 ± 0.82^a, b, c^	8.68 ± 0.22^a^	6.56 ± 0.11^b^
Sapindus saponaria	46.93 ± 0.24^ c^	11.74 ± 0.32^b^	10.64 ± 0.22^c^
*Eucalyptus globulus* (leaves)	165.76 ± 0.13^f^	10.23 ± 0.12^b^	18.34 ± 0.13^d^
*Annona muricata* (leaves)	40.21 ± 0.15^a, b^	9.48 ± 0.64^a^	6.90 ± 0.21^b^
*Abelmoschus esculentus* (leaves)	36.86 ± 0.25^a^	8.96 ± 0.08^a^	5.54 ± 0.17^a, b^
Butylhydroxytoluene	426.85 ± 0.11^g^	63.89 ± 0.97^d^	52.43 ± 0.75^e^

Antioxidant activities of plant extracts

The antioxidant activity was evaluated by determining the DPPH free radical scavenging activity as well as the FRAP of the extracts (Table 4). It emerges from the results obtained that, among the tested extracts, vitamin C recorded the smallest EC_50_ value (i.e., corresponding to the greatest DPPH free radical scavenging activity) compared to the *Kotschya strigosa* and *K. pinnata *extracts (Table 4). Furthermore, *K. strigosa* and *K. pinnata* extracts recorded the smallest FRAP compared to vitamin C (Table 4).

**Table 4:** Antioxidant activities (EC50 and FRAP) of extracts from Kotschya strigosa and Kalanchoe pinnata Each value represents the mean ± standard deviation of three repetitions; in the same column, the values ​​assigned different letters (a-b) are significantly different at the 5% probability level according to one-way ANOVA and the Waller-Duncan test DPPH: diphenyl-1-picrylhydrazyl; EC_50_: equivalent concentrations of test samples scavenging 50% of DPPH radical; FRAP: ferric reducing antioxidant power

Samples	DPPH, EC_50_ (µg/mL)	FRAP (mmol FeSO_4_/g)
*Kotschya strigosa* (G2)	2.24 ± 0.56^a^	142.67 ± 0.58^a^
*Kalanchoe pinnata* (G3)	2.20 ± 0.59^a^	142.58 ± 0.63^a^
Vitamin C	1.36 ± 0.13^b^	156.85 ± 0.55^b^

Antibacterial activity of the plant extracts

The antibacterial activity of the plant extracts was evaluated through the determination of the MICs and the MBCs against Gram-positive and negative bacteria (Table 5). The analysis of the results showed that the antibacterial activity varied according to the plant and bacterial species tested (MIC = 64-2048 μg/ml). The smallest MIC values (i.e., corresponding to the greatest antibacterial activities) were obtained with the *K. strigosa* and *K. pinnata* extracts against *K. pneumoniae* 106, whereas most of the MBC values were obtained with these extracts, suggesting their bactericidal effects. The other extracts did not show bactericidal activity to a considerable extent. The extract of *K. strigosa* (CMI = 64-1024 µg/ml) was the most active, followed in ascending order by those of *K. pinnata*, *Annona muricata*, *A. esculentus*, *P. umbellatum*, *S. *​​​​*saponaria*, *E. globulus,* and *Eupatorium cannabinum*. In comparison to the plant extracts, the smallest MIC values (i.e., corresponding to the greatest antibacterial activities) were recorded with Augmentin on *K. pneumoniae* 106. The degree of sensitivity of the bacterial species and hence antibacterial activity of test samples is of the order *P. aeruginosa* 21 > *K. pneumoniae* 106 > *K. pneumoniae *22 > *K. pneumoniae* ATCC 10031 > *S. pneumoniae* ATCC 49619 > *K. pneumoniae* 46 > *K. pneumoniae* 56 > *K. pneumoniae* 26 > *Klebsiella* *oxytoca* 26 > *K. pneumoniae* 44 > *K. oxytoca* 43 > *K. oxytoca* 107 > *K. pneumoniae* 31, as shown by the MICs. The activities of the extracts were lower than those of amoxicillin and Augmentin, used as reference antibiotics.

**Table 5:** Antibacterial activity (MIC and MBC in µg/mL) of the different plant extracts according to the tested bacteria MIC: minimum inhibitory concentration; MBC: minimum bactericidal concentration; -: not determined

Bacteria	*Piper umbellatum*		*Eupatorium cannabinum* (leaves)		Sapindus saponaria		*Eucalyptus globulus *(leaves)		*Annona muricata *(leaves)		*Abelmoschus esculentus *(leaves)		*Kalanchoe pinnata *(leaves)		*Kotschya strigosa*		Augmentin		Amoxicillin	
	MIC	MBC	MIC	MBC	MIC	MBC	MIC	MBC	MIC	MBC	MIC	MBC	MIC	MBC	MIC	MBC	MIC	MBC	MIC	MBC
*Pseudomonas aeruginosa* 21	256	256	1024	-	512	1024	512	512	128	512	1024	1024	256	256	512	256	128	128	128	-
*Klebsiella pneumoniae* 22	2048	-	2048	-	512	-	1024	-	1024	-	512	1024	512	1024	128	512	16	64	64	128
*Klebsiella pneumoniae* 26	-	-	2048	-	512	2048	2048	-	-	-	2048	-	512	1024	1024	-	64	64	64	128
*Klebsiella pneumoniae* 31	-	-	-	-	-	-	2048	-	2048	-	-	-	1024	-	512	1024	128	256	-	-
*Klebsiella pneumoniae* 44	2048	-	-	-	1024	-	1024	-	-	-	-	-	128	512	1024	2048	16	32	64	128
*Klebsiella pneumoniae* 46	-	-	-	-	2048	-	512	2048	1024	1024	-	-	1024	2048	512	1024	16	64	128	128
*Klebsiella pneumoniae* 56	2048	-	2048	-	1024	-	1024	-	1024	-	2048	-	512	1024	512	2048	64	128	256	-
*Klebsiella pneumoniae* 106	-	-	1024	-	1024	-	512	-	128	512	128	1024	64	128	64	512	8	32	64	256
*Klebsiella oxytoca* 26	1024	-	512	1024	-	-	2048	-	1024	-	-	-	512	1024	1024	2048	16	32	256	-
*Klebsiella oxytoca *43	1024	-	1024	2048	2048	-	2048	-	2048	-	-	-	1024	-	512	1024	256	-	256	-
*Klebsiella oxytoca* 107	1024	1024	2048	-	-	-	1024	-	1024	2048	-	-	512	2048	1024	-	256	-	-	-
*Streptococcus pneumonia*e ATCC 49619	512	-	-	-	1024	-	2048	-	1024	1024	1024	-	512	512	512	1024	32	128	64	128
*Klebsiella pneumoniae* ATCC 10031	512	2048	-	-	2048	-	512	512	-	-	512	1024	128	1024	512	512	64	128	128	256

Effect of the combination of the extract and Augmentin

The effect of the combination of *K. strigosa* extract and Augmentin was studied, and the results are depicted in Table 6. *K. strigosa* extract (leaves) and Augmentin exert in association an additive effect against *P. aeruginosa* 21, *K. pneumoniae* 44, *K. pneumoniae* 46, *K. pneumoniae* 56, *K. pneumoniae* 106, *K. oxytoca* 26, and *K. oxytoca* 43; a synergistic effect against *K. pneumoniae* 22,* K. oxytoca* 107, *S. pneumoniae* ATCC 49619, and *K. pneumoniae* ATCC 10031; and an indifference effect against *K. pneumoniae* 26 and *K. pneumoniae* 31 (Table 6).

**Table 6:** Fractional inhibitory concentrations (FICs) calculated for the combination of Augmentin and Kotschya strigosa extract (leaves) against the different bacteria studied ∑ FIC: sum of fractional inhibitory concentrations

Bacteria	∑ FIC	Interpretation
*Pseudomonas aeruginosa* 21	0.75	Additive
*Klebsiella pneumoniae* 22	0.31	Synergy
*Klebsiella pneumoniae* 26	1.00	Indifference
*Klebsiella pneumoniae* 31	1.50	Indifference
*Klebsiella pneumoniae *44	0.62	Additive
*Klebsiella pneumoniae* 46	0.75	Additive
*Klebsiella pneumoniae* 56	0.62	Additive
*Klebsiella pneumoniae* 106	0.53	Additive
*Klebsiella oxytoca* 26	0.62	Additive
*Klebsiella oxytoca* 43	0.75	Additive
*Klebsiella oxytoca* 107	0.37	Synergy
*Streptococcus pneumoniae* ATCC 49619	0.09	Synergy
*Klebsiella pneumoniae* ATCC 10031	0.28	Synergy

Mechanism of antibacterial activity

*Effect of the *K. strigosa* Extract on the Outer Membrane Permeability*

**Figure 1:**
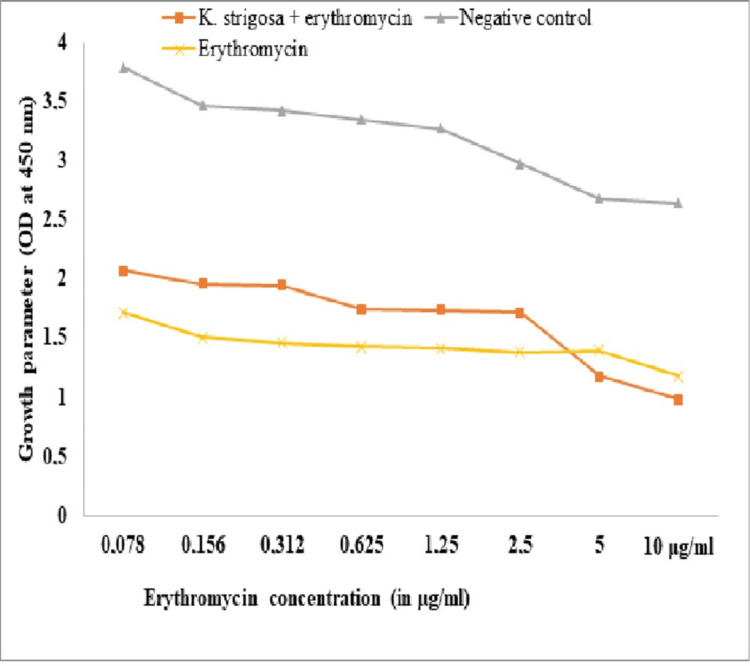
Effect of the combination of the Kotschya strigosa extract and erythromycin on the growth of Klebsiella pneumoniae 106 according to erythromycin concentrations The results are expressed as mean ± standard deviation of three repetitions. *K. strigosa*: *Kotschya strigosa*; OD: optical density

The co-incubation of erythromycin with bacterial suspension had a measurable inhibitory effect on the *K. pneumoniae* growth (Figure 1). The potentiating effect of the *K. strigosa* extract on the membrane permeability to erythromycin was not observed against *K. pneumoniae *106 at the concentrations of erythromycin less than 2.5 µg/mL, where the effect of the erythromycin + *K. strigosa* extract was less than that of the erythromycin alone (Figures 1). Meanwhile, the effect of the combination of the *K. strigosa* extract with erythromycin on the outer membrane permeability was higher than that of the erythromycin alone on *K. pneumoniae* 106 at the tested concentrations of 5 µg/mL and 10 µg/mL (Figure 1).

*Effect of the *K. strigosa* Extract on the Growth Kinetics of *K. pneumoniae

**Figure 2:**
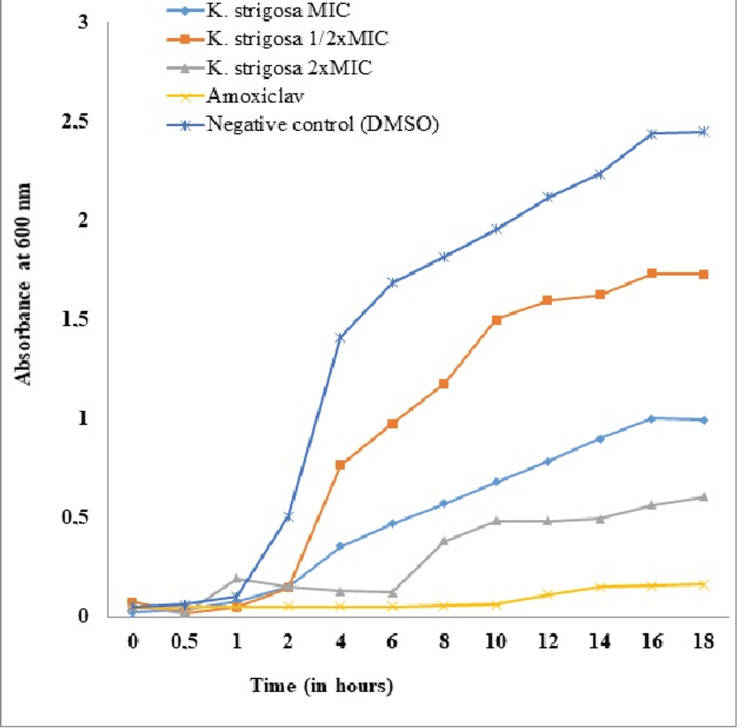
Effect of the Kotschya strigosa extract on the growth kinetics of Klebsiella pneumoniae 106 DMSO: dimethyl sulfoxide; MIC: minimum inhibitory concentration; *K. strigosa*: *Kotschya strigosa*

The growth kinetic curves of *K. pneumoniae* in the absence of any treatment (DMSO) and in the presence of Augmentin and *K. strigosa *extract (at MIC, MIC/2, and 2MIC concentrations) are shown in Figure 2 to highlight the influence that the plant extract can have on the kinetics of bacterial growth. It appears that the curve of the inoculum in the presence of DMSO presents four phases (latency phase, acceleration phase, exponential phase, and stationary phase) with a final OD of 2.4. In the presence of the plant extract at different concentrations (MIC, MIC/2, and 2MIC), a considerable decrease in cell growth is observed with a lower final OD for the concentrations of MIC and 2MIC compared to MIC/2 for the plant extract. This extract presents all the phases of bacterial growth: a lag phase (0-2 hours), an acceleration phase (2-8 hours), an exponential phase (8-16 hours), and a stationary phase (16-18 hours). No considerable growth is observed in the presence of Augmentin; hence, an almost constant lag phase until the end of the experiment (Figure 2).

*Effect of the *K. strigosa* Extract on the Enzymatic Activity of Dehydrogenases of the Respiratory Chain of *K. pneumoniae

**Figure 3:**
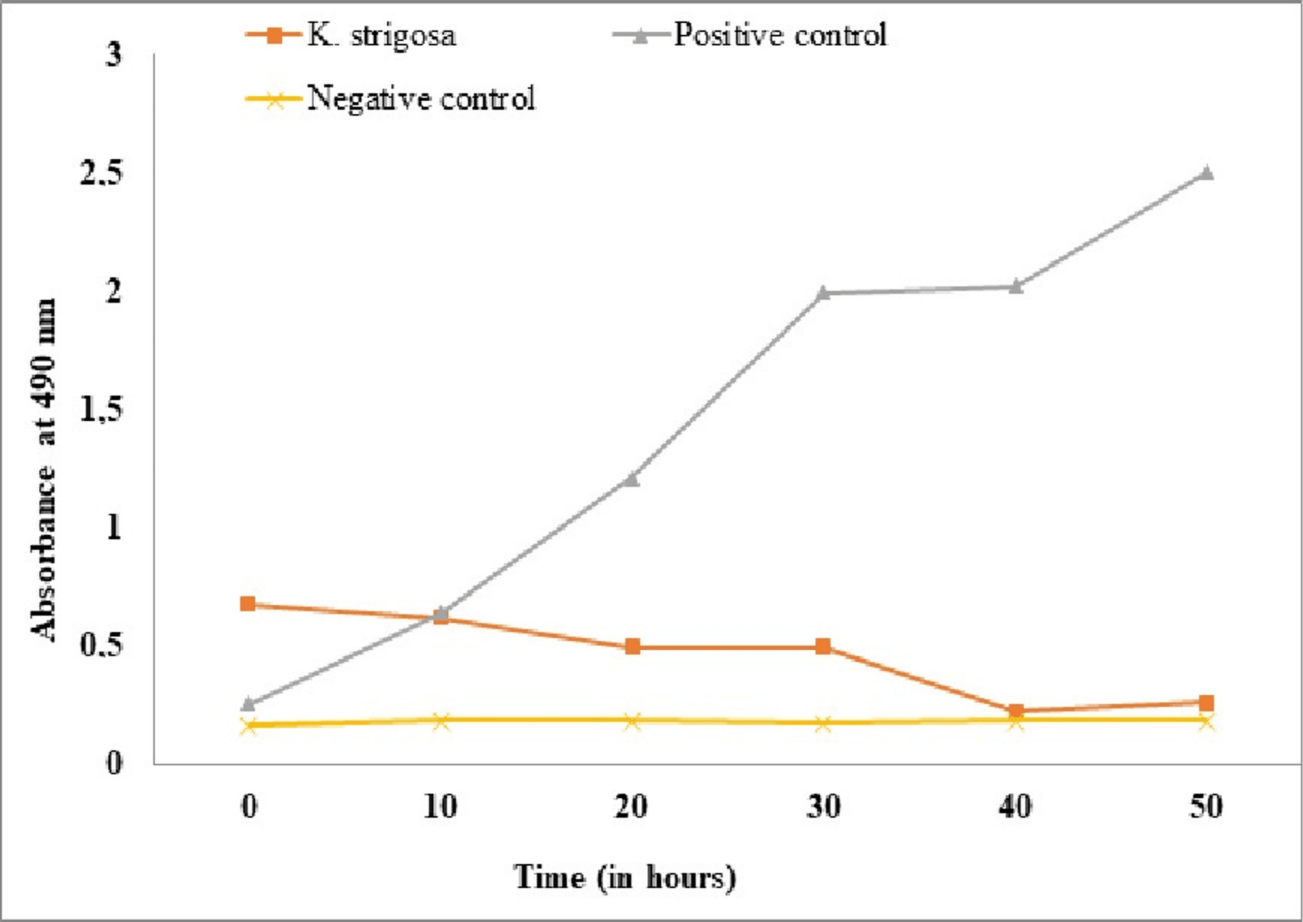
Effect of the Kotschya strigosa extract on the activity of respiratory chain dehydrogenases of Klebsiella pneumoniae 106 as a function of time *K. strigosa*: *Kotschya strigosa*

Figure 3 shows the effect of *K. strigosa *extract on dehydrogenases of the respiratory chain of *K. pneumoniae*. The activity of respiratory chain dehydrogenases in untreated healthy bacterial cells (positive control) increased with incubation time, while the activity of respiratory chain dehydrogenases underwent virtually no change in tubes containing cells heated at 100°C for 20 minutes (negative control). The enzymatic activity of bacterial cells treated with the 64 μg/ml extract remained significantly lower than that of the positive control during the 40-minute incubation.

*Effect of the *K. strigosa* Extract on ATPases/H+ Proton Pumps of *K. pneumoniae

**Figure 4:**
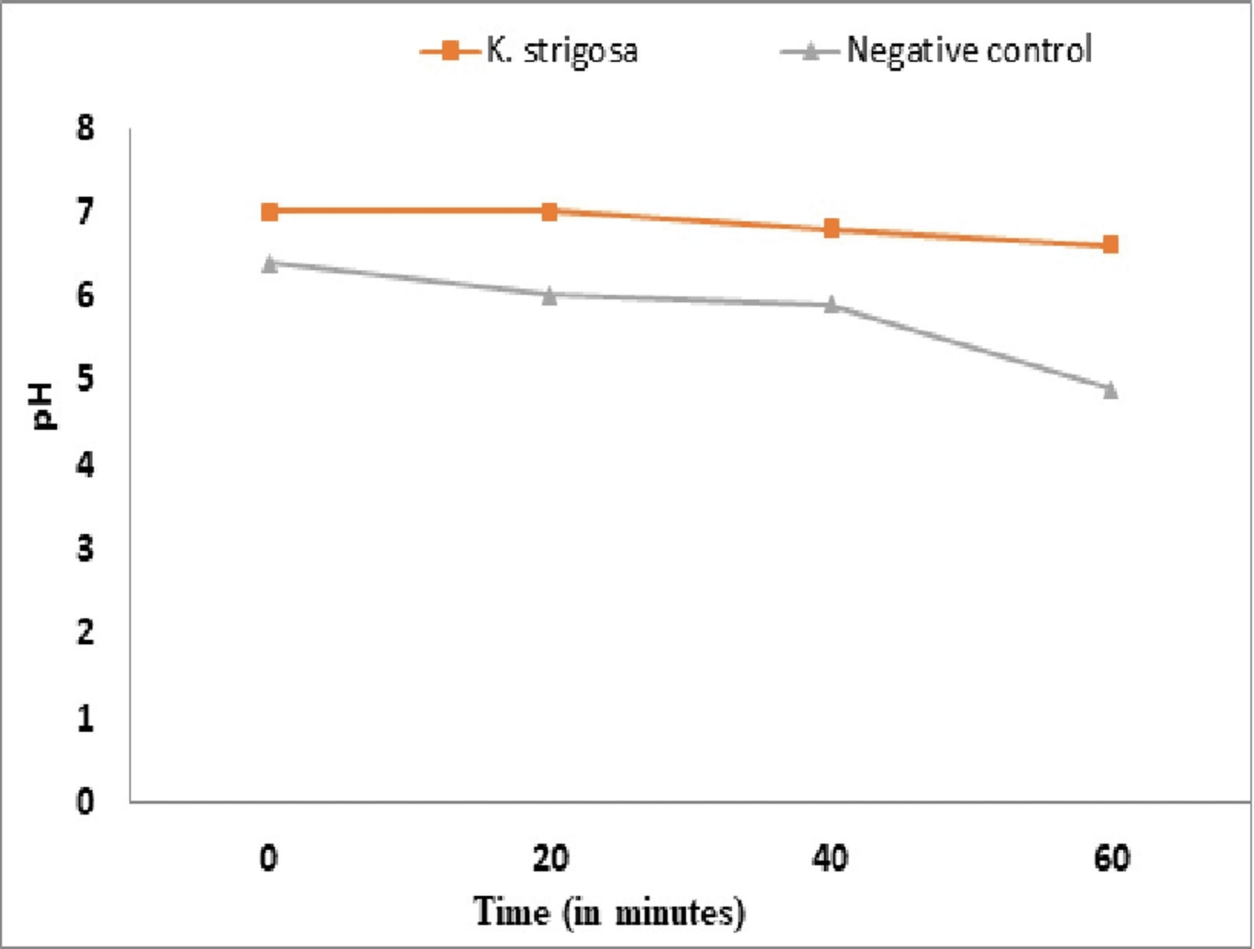
Effect of the Kotschya strigosa extract on Klebsiella pneumoniae proton pumping as a function of time *K. strigosa*: *Kotschya strigosa*

Figure 4 shows the pH variation curves as a function of the time of the reaction medium containing *K. pneumoniae* cultures treated with the extract of *K. strigosa*. It appears that the pH values of the medium slightly decreased during the first 20 minutes with the extract compared to the control. After 40 minutes, the pH decreased significantly (p < 0.05). The effect of the extract remained significantly higher compared to that of the negative control.

*Effect of the *K. strigosa* Extracts on the Nucleotide Leakage of *K. pneumoniae

**Figure 5:**
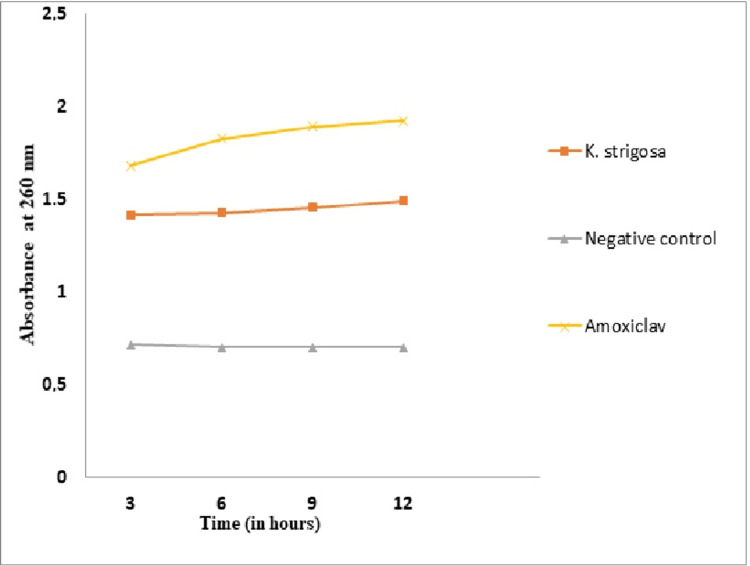
Evolution of Klebsiella pneumoniae nucleotide leakage as a function of incubation time with Kotschya strigosa extract *K. strigosa*: *Kotschya strigosa*

A significant leakage of nucleotides is observed from the bacteria treated with *K. strigosa* extract as a function of incubation time compared to the negative control (Figure 5). The effect observed with the *K. strigosa* extract is greater than that of Augmentin irrespective of the incubation time.

## Discussion

Medicinal plants are an undeniable source of effective natural substances that can help combat recalled human pathologies such as microbial, parasitic, and viral infections and cancer phenotypes. The differences in sensitivity observed between different bacterial species for the same extract may be due to genetic variability in strain resistance [13]. During the determination of MIC and MBC, it was noted that most of the extracts had MBC/MIC ratios > 4 μg/ml; this indicates that these extracts generally have a bacteriostatic effect [13].

The results of this study show that all our extracts contain phenols, flavonoids, steroids, tannins, and triterpenes, the content of which varies depending on the extract and the part used. These compounds are known for their antimicrobial activity, although initially produced by plants as a means of defense against diseases. The presence of phenols in all plant extracts is not surprising because they are present in all vascular plants. Phenolic compounds from plants constitute powerful antioxidants in humans, capable of preventing oxidative damage to biomolecules such as DNA, lipids, and proteins that play a role in chronic diseases, and it has already been demonstrated the linear relationship that exists between the composition of phenolic compounds and the antioxidant power of plants that act through their hydroxyl groups [17]. The results of this study corroborate those of Roersch [18], who highlighted the presence of flavonoids, steroids, terpenes, and alkaloids in the extract of *P. umbrellatum*, and are almost identical to those of Gueddah and Soualat [19], who showed the presence of phenolic compounds and flavonoids but the absence of tannins in the extracts of *K. pinnata *and *E. globulus*. The results obtained following the various antioxidant tests (DPPH and FRAP) reveal the activity of certain plant extracts tested, and these extracts also showed the presence of phenolic compounds in high content; this indicates that the more a plant contains phenolic compounds, the better its antioxidant activity. Phenolic compounds have been identified as hydrogen donors and therefore very good antioxidants [15]. This could also be due to the presence of other types of secondary metabolites detected in the plant or their synergistic actions responsible for their reducing properties. The reducing properties are also associated with the presence of reducers, whose antioxidant action has been demonstrated by reducing chain reactions by the gain of a hydrogen atom [15]. These results corroborate those of Sokoudjou et al. [20], who showed both antisalmonella and antioxidant activities of *Curcuma* *longa* and *Canarium* *schweinfurthii *extracts.

Antibiotic combinations can lead to synergistic effects, especially during the therapy of bacterial infections. These combinations have been recognized as being able to delay the emergence of resistant strains of microorganisms [14]. The synergistic effect between plant-derived compounds and antibiotics makes it possible to use antibiotics when the efficacy of the latter alone is reduced. These observations could explain the evaluation of the antibacterial activity of the combination of *K. strigosa* extract and Augmentin because, in addition to substances with direct antibacterial activity, it has been shown that within plants, other substances can act as adjuvants by modulating the activity of antibacterial agents [21]. The effect of the combination of the plant extracts with conventional antibiotics could also be useful in combating bacterial drug resistance [14].

The outer membrane is a barrier allowing selective permeability in nutrient penetration to support bacterial growth [15]. Measuring the permeability of the bacterial membrane is fundamental for the study of antimicrobials. Thus, antibiotics such as erythromycin at low concentrations are not able to penetrate the intact outer membrane of Gram-negative bacteria [15] because of their large sizes. However, this antibiotic crosses the damaged outer membrane of bacteria at low concentrations [15]. In this study, *K. strigosa* extract resulted in a decrease in bacterial growth at concentrations below the MIC of erythromycin. This may reflect the ability of these extracts to facilitate the passage of erythromycin through the bacterial outer membrane through membrane destabilization leading to increased membrane permeability. This damage to the bacterial cell membrane would be the origin of the anti-Klebsiella activity of *K. strigosa*. Similar effects were observed by Ekom et al. [15] with *Capsicum annuum *extract on *P. aeruginosa *PA01, *Escherichia coli* 64R, and *Staphylococcus aureus* 18.

A significant shortening of the exponential growth phase of* K. pneumoniae* in the presence of the *K. strigosa *extract was observed. The results obtained, in particular the considerable decrease in the final OD of the growth curve in the presence of the extracts, corroborate a previous study [22] that revealed that this decrease is due to stress conditions where growth is slowed down following the destruction of certain bacteria, hence the denaturation of enzymes, transport systems, and other proteins. The inhibition of bacterial growth in the presence of Augmentin highlights the bacteriostatic activity of the latter. Furthermore, a longer latency phase is observed in the presence of the extract compared to the control containing DMSO; this may be due to the partial deactivation of the cells reducing the initial vital load. The bacteria need more time and newly synthesized proteins and enzymes to be able to divide. This could justify the inhibition of the growth of the extract of* K. strigosa*, particularly in the latency phase.

All substances acting on the dehydrogenases of the respiratory chain located in the internal membrane of the bacteria, which is the site of energy production, must first cross the external membrane to reach the dehydrogenases [15]. Thus, the activity of the respiratory dehydrogenase in the bacteria treated with the *K. strigosa* extract was lower than that of the positive control, which indicates that the bacterial electron transport chain was affected or even inhibited by the extract. These substances could inhibit bacterial growth by uncoupling the electron transport chain of bacteria. This uncoupling can either inhibit respiratory chain dehydrogenases by disrupting oxidative phosphorylation or destroy them with the consequence of inhibiting cell respiration [15]. These results are similar to those of Ekom et al. [15], who showed that *C. annuum* fruit extract decreases the activity of respiratory chain dehydrogenases of bacteria.

H^+ ^ATPase proton pumps are involved in the regulation of bacterial cytoplasmic pH and the supply of energy in the form of ATP to the bacteria. Both are necessary for bacterial growth. The antibacterial power of many compounds is justified by their interference with bacterial proton pumping [23]. *K. pneumoniae* has an optimal growth pH between 6 and 8 [24]. According to the results obtained, the decrease in the pH of the medium containing the bacterial cells treated with the extract and the control reflects the inhibition of proton pumping of *K. pneumoniae*. H^+^ ATPase proton pumps would be the target of the action of the extract of *K. strigosa*. These results show that some metabolites contained in this plant extract have the ATPase-H^+^ proton pumps as their target of action. These findings are consistent with those previously reported [15].

A leakage of cytoplasmic molecules such as nucleotides, proteins, and reducing sugars is observed due to the action of antibacterial agents during major damage to the cytoplasmic membrane. These are released after membrane rupture, and their quantification is done by monitoring their absorbance. The addition of the extract of *K. strigosa* resulted in a significant leakage of nucleotides in bacteria. This leakage could reflect the loss of bacterial membrane integrity and, consequently, the death of the bacteria. The leakage of intracellular materials from cells is an indicator of major damage to the cell membrane [15]. Similar results were noted by Ekom et al. [15] with *C. annuum* extract on *E. coli*, *P. aeruginosa*, and *S. aureus*.

Limitations

All these results encourage further investigations to isolate and quantify the bioactive compounds from the most active plant to improve our understanding of possible antibacterial and antioxidant activities. Also, the possibility remains that sites of action other than the cytoplasmic membrane exist. So, further works including in vivo studies are warranted.

## Conclusions

In the present study, the antibacterial activity, antioxidant potential, synergistic effect, phytochemical content, and mechanisms of antibacterial action of the selected Cameroonian medicinal plants were evaluated. The results of this study demonstrated the antibacterial and antioxidant properties of the tested plants, which can be linked to their total phenolic, flavonoid, sterol, triterpene, and tannin contents. *K. strigosa *leaf extract was the most active tested sample and exerted, with Augmentin, additive, synergistic, and indifferent effects depending on bacterial species. The antibacterial activity of *K. strigosa* extract possibly arises as a result of the shortening of the exponential growth phase, longer latency phase, and perturbation of the bacteria membrane, which then causes the leakage of intracellular materials, incapacity of the membrane to regulate the internal pH, and inactivation of the respiratory chain of dehydrogenase. The findings of the present study support the traditional use of the selected medicinal plants in the treatment of infectious diseases caused by the tested bacteria.
